# Composite Proton Exchange Membrane Based on Poly-1-Vinyl-1,2,4-Triazole with Sulfofullerene

**DOI:** 10.3390/polym17233171

**Published:** 2025-11-28

**Authors:** Ruslan Usmanov, Artem Emel’yanov, Nadezhda Kuznetsova, Tatyana Semenova, Dmitriy Chepenko, Galina Prozorova, Alexander Pozdnyakov

**Affiliations:** A.E. Favorsky Irkutsk Institute of Chemistry of the Siberian Branch of the Russian Academy of Sciences, 1 Favorsky Street, 664033 Irkutsk, Russia

**Keywords:** poly-1-vinyl-1,2,4-triazole, polyhydroxysulfonated fullerene C_60_, nanocomposite, proton exchange membrane

## Abstract

Proton exchange membrane fuel cells are environmentally friendly, safe clean energy devices that have the potential to change the world. Proton exchange membrane fuel cells are a promising replacement for traditional power generation devices. Nanocomposite proton exchange membranes have high energy efficiency, which allows them to be considered as a new generation of proton exchange materials. This paper presents for the first time the synthesis and properties of nanocomposite proton exchange membranes based on poly-1-vinyl-1,2,4-triazole modified with polyhydroxysulfonated fullerene. Sulfofullerene intercalated into the polymer matrix improves all key membrane properties. The PEM nanocomposites exhibit a proton conductivity of up to 1.67 mS/cm and a uniform distribution of carbon nanoparticles of up to 10 nm in size. It was established that high dispersion and stabilization of nanoparticles are ensured by the acid–base interaction of sulfofullerene with the heterocycles of the polymer matrix. Stabilization of functionalized fullerenes by a matrix of semi-interpenetrating polymer networks is an innovative approach for creating nanocomposite proton-conducting systems. The obtained fullerene-containing PEMs demonstrate a high potential for wide practical application in various fuel cells.

## 1. Introduction

Energy-efficient technologies reduce dependence on limited fossil fuels and strengthen global energy security. Particular attention of researchers is directed to the development of hydrogen energy, including proton exchange membrane fuel cells (PEMFCs) [1,2]. The proton exchange membrane (PEM) is key component determining the efficiency of PEMFC [3,4,5]. Therefore, the research and development of new proton exchange membranes is in demand to meet the growing industrial demand for PEMFCs. Recently, nanocomposite PEMs modified with carbon nanomaterials, such as fullerenes, nanotubes, and graphene, have been increasingly studied. [6,7,8,9,10,11,12,13,14]. Fullerenes are a unique type of carbon molecule that stands out due to their distinctive spherical shape [15,16,17,18,19,20]. Compared to other carbon nanomaterials, fullerenes are easily soluble in organic solvents and can be easily functionalized, making them versatile modifiers for various PEMs [21,22,23]. Research devoted to the production of fullerene-containing PEMs using various fullerenes and their derivatives is widely known [24,25,26,27,28]. Among them, the fullerene functionalized with proton-active sulfonic groups, i.e., polyhydroxysulfonated fullerene (PHSF), is the most promising [29]. It is known that the sulfofullerene granules exhibit intrinsic proton conductivity in the range from 10^−3^ to 10^−2^ S/cm [30,31,32]. The proton conductivity of sulfofullerenes is provided by the small distance between sulfonic groups (less than 2.5 Å) in the structure of particles with a face-centered cubic crystal lattice [33,34]. This allows protons to jump between functional groups according to the Grotthuss mechanism [35,36].

PEMs based on perfluorosulfonic acid exhibit high proton conductivity up to 10^−2^ S/cm only under high humidity conditions [37]. There is a study on the use of sulfofullerenes to improve the properties of perfluorinated PEM, such as Nafion [38]. The introduction of 1 wt.% sulfofullerene increased the proton conductivity of the membranes from 5.45 × 10^−2^ to 7.24 × 10^−2^ S/cm. At the same time, an increase in ion exchange capacity (IEC) and a decrease in water uptake are observed. Sulfofullerenes are included in the membrane structure in the form of nanoparticles with an average size of 50 nm. It is known that the fluorocarbon skeleton of Nafion exhibits high fluorophilicity, which reduces its chemical affinity with non-fluorinated organic molecules [39,40]. Thus, the uneven distribution of large nanoparticles in the Nafion-type PEM composite composition is associated with a low chemical affinity for sulfofullerene [41]. The development of composite PEMs based on other polymer systems is a sought-after area of modern research.

Polyvinylazoles are promising materials for creating PEMs that can conduct protons under low humidity conditions [42]. Proton transfer in systems based on them is ensured by the protonation and deprotonation processes of nitrogen-containing heterocycles and reaches proton conductivity values of up to 1.2 × 10^−3^ S/cm [43,44,45,46]. Among polyvinylazoles, poly-1-vinyl-1,2,4-triazole (PVT) has wide practical significance [47]. PVT is used to develop noble metal sorbents [48], biocompatible hydrogels [49], functional organosilicon coatings [50], polymer nanocomposites [51,52], luminescent complexes [53] and proton exchange membranes [54]. In some studies, PVT is referred to as a “catalyst” for proton conductivity due to its ability to form low-barrier hydrogen bonds [55,56]. Due to the presence of a “pyridine” nitrogen atom, PVT exhibits a high complexation ability [57]. The combination of these properties in PVT allows it to be considered as a promising polymer base for the formation of new nanocomposite PEMs.

One of the significant problems associated with composite PEMs containing carbon nanomaterials is the formation of large particles with a non-uniform distribution within the membrane structure [29,35,58]. Various functionalized fullerenes also tend to form large aggregates in solutions. The formation of large particles in the PEM structure decreases in the main membrane characteristics, including proton conductivity [28,38,59]. Thus, the efficiency of fullerene-containing PEMs, as well as the size and uniformity of nanoparticle distribution, is largely determined by the polymer matrix stabilizing ability.

In this work, the synthesis of new nanocomposite PEMs based on a PVT modified PHSF is presented for the first time. Using modern analytical methods, the main characteristics of fullerene-containing PEMs were determined, including proton conductivity, thermal stability, activation energy of proton transport, and water uptake structure and morphology. The key principles of the interaction of PHSF nanoparticles with the PVT matrix were studied. Possible mechanisms of proton conductivity and their dependence on the size and distribution pattern of PHSF nanoparticles are analyzed in detail. The obtained data demonstrate the high potential of using fullerene-containing PEMs in fuel cells.

## 2. Materials and Methods

### 2.1. Materials

The following materials and compounds were used in the work: The initial 1-vinyl-1,2,4-triazole (VT) was used as the initial compound for obtaining PVT, which was synthesized and purified according to the known procedure: b.p. 48–50 °C (2–3 mm Hg), n_D20_ 1.5100 [60]. 1-methyl-2- pyrrolidone (MP, 99.5%) as a solvent, a,a-azobisisobutyronitrile (AIBN) as an initiator, and diethyl ether were purchased from Sigma-Aldrich (Munich, Germany) and distilled over calcium hydride immediately before use. As-received fullerene C_60_ with 99.95% purity was obtained in our own production from A.E. Favorsky Irkutsk Institute of Chemistry (Irkutsk, Russia) was used to synthesize sulfofullerene. Concentrated H_2_SO_4_ (oleum), acetonitrile, 3% H_2_O_2_, FeSO_4_, NaOH, phenolphthalein, HCl, polyvinyl alcohol (PVA), and oxalic acid (OA) were purchased from Sigma-Aldrich (Munich, Germany) and used without purification.

### 2.2. Methods

#### 2.2.1. Characterization

Fourier transform infrared with attenuated total reflectance (FTIR-ATR) spectra were recorded on a Spectrum Two FT-IR spectrometer (PerkinElmer, Waltham, MA, USA) in the wavenumber range 400–4000 cm^−1^ with a thin film sample. Elemental analysis was performed using an analyzer Flash EA 1112 Series (Thermo Fisher Scientific, Cambridge, UK). The molecular weight and molecular weight distribution of the samples were determined by gel penetration chromatography on a Shimadzu LC-20 Prominence system equipped with a Shimadzu RID-20A differential refractometric detector (Shimadzu Corporation, Kyoto, Japan). The flow rate was 1 mL/min. The samples were weighed and dissolved in DMF at room temperature for 24 h with stirring. The solution concentration was 10 mg/mL. Calibration was performed using a series of polystyrene standards, Polystyrene High EasiVials (PL2010-0201), which contained 12 samples with a molecular weight of 162 to 6,570,000 g/mol. The surface morphology of sulfofullerenes and composite membranes was examined using a scanning electron microscope (SEM) FEI Quanta 200 (FEI Company, Hillsboro, OR, USA) combined with EDAX X-ray microanalysis attachment GENESIS XM 2 60-Imaging SEM with APOLLO 10 (FEI Company, Hillsboro, OR, USA). Micrographs of composite membranes, particle size, and distribution patterns were studied using a transmission electron microscope Leo 906E (Carl Zeiss AG, Oberkochen, Germany). The hydrodynamic particle sizes were determined using the dynamic light scattering method (DLS) (Brookhaven Instruments Corporation, Holtsville, NY, USA). Measurements were performed in thermostated cuvettes at an operating temperature of 25 °C and a scattered light detection angle of 90.0°. Solutions with a concentration of 0.1 mg/mL were prepared for the measurements using MP as the solvent.

The electrical conductivity of the membranes was determined by impedance spectroscopy using a PARSTAT 2273 electrochemical workstation (Princeton Applied Research, Oak Ridge, TN, USA). Measurements were carried out in C/membrane/C cells in the frequency range of 1 Hz–1 MHz at a relative humidity of 12%. The electrical conductivity data at temperatures of 40–80 °C were used to calculate the proton transport activation energy (E_a_). The thickness of the membranes was measured at five points at the corners and in the middle of the sample, using a IDC-125EB micrometer (Mitutoyo, Kawasaki, Japan).

To determine water uptake, membrane samples with different dopant contents were dried in a vacuum oven at 65–70 °C for 24 h. After drying, the membranes were cooled to room temperature and weighed on an analytical balance to determine their dry mass (m_0_). Film samples were placed in weighing bottles, filled with deionized water, and kept in a thermostat at 30 °C for 8 h. The membranes were then removed from the water, blotted with filter paper to remove excess moisture, and reweighed. The resulting mass (m_1_) is the mass of the sample after contact with water. The water absorption results were expressed as a percentage of the dry sample mass. The water uptake (WU, %) was calculated using the following formula:

(1) $$WU=\frac{\left(m_{1}-\;m_{0}\right)}{m_{0}}\times 100\%$$

To assess the possible leaching of PHSF from the membrane structure, an acid leaching test was carried out according to a well-known method [61]. The membrane samples were immersed in deionized water for 2 h. The samples were removed, dried, and weighed every 30 min. The residual mass of PHSF in the membranes was calculated using the following formula:

(2) $${RW}_{PHSF}=\frac{1-(m_{0}-\;m_{n})}{m_{PHSF}}\times 100\%$$

where m_0_—is the initial mass of the composite PEM, m_n_—is the mass of the membrane after leaching for n h, and m_PHSF_—is the initial mass of the functionalized fullerene in the membrane.

The degree of membrane swelling was determined by measuring the dimensions of 10 mm × 10 mm film samples. The samples, previously dried in a vacuum oven to constant weight and placed in a water container at 25 °C for 24 h. The film was then removed from the water, excess water was removed with filter paper, and the dimensions were measured after swelling. The percentage change in dimensions was calculated using the following formula:

(3) $$\Delta l=\frac{\left(l_{1}-\;l_{0}\right)}{l_{0}}\times 100\%$$

where l_0_—dimensions of the original PEM composite sample, l_n_—size of the swollen membrane sample.

To determine the oxidative stability of composite membranes, film samples measuring 10 mm × 10 mm, dried to constant weight, were placed in a container containing Fenton’s reagent with constant stirring at 60 °C. The oxidative stability was assessed by recording the residual weight and exposure duration before membrane decomposition.

#### 2.2.2. Synthesis of Poly-1-Vinyl-1,2,4-Triazole

**Scheme 1:**
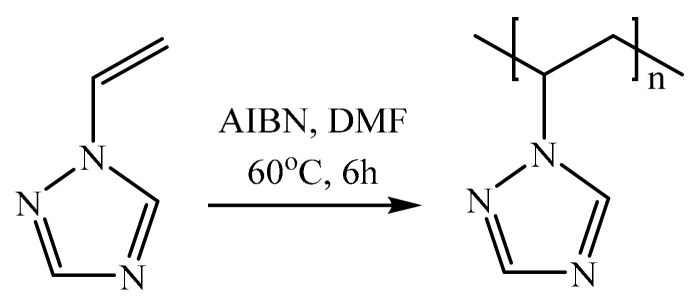
Polymerization of VT.

**Figure 1:**
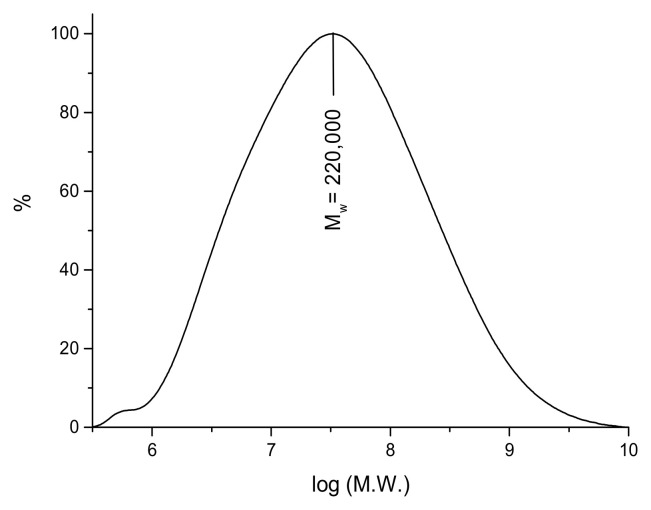
Molecular weight distribution of poly-1-vinyl-1,2,4-triazole.

PVT was obtained by the radical polymerization of VT in DMF in the presence of 1 wt.% AIBN in argon at 60 °C for 6 h (Scheme 1). The resulting viscous mass was diluted with DMF, precipitated twice into a mixture of ethanol and acetone (1:2), and dried in P_2_O_5_ to a constant mass at 50 °C. The polymer is a white powder, thermally stable up to 330 °C, soluble in H_2_O, DMF, DMSO, and MP. The product yield was above 83%. The molecular weight of the synthesized PVT was determined using gel permeation chromatography. The average molecular weight is Mw 220,000 Da (Figure 1).

The sample showed a unimodal molecular weight distribution, and the polydispersity coefficient was 1.91 (Figure 1). FTIR (ν, cm^−1^): 3110 (C–H), 2928 (CH_2_), 1507 (C=N), 1436 (C–N), 1276 (N–N), 1139 (C–H), 1005 (C–H), and 659 (C–N). ^1^H NMR (D2O, δ, ppm): 8.09–7.50 (br m, 2H, triazole ring), 4.11–2.70 (br m, 1H, CH in the polymer main chain), 2.30–1.75 (br, 2H, CH2 in the polymer main chain); ^13^C NMR (D2O, δ, ppm):152.6–150.9, 143.4–141.9 (CH, triazole ring), 57.0–53.7 (CH in the polymer main chain), 37.7–35.3 (CH2 in the polymer main chain).

#### 2.2.3. Synthesis of Polyhydroxysulfonated Fullerene C_60_

**Scheme 2:**
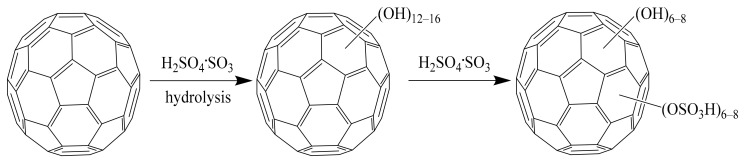
Synthesis of polyhydroxysulfonated fullerene C_60_.

Polyhydroxysulfonated fullerene C_60_ (PHSF) was synthesized according to a known methodology [62] (Scheme 2). Fullerene C_60_ was exposed to concentrated sulfuric acid (oleum) at 60 °C in an inert N_2_ atmosphere for 36 h. The obtained polycyclosulfated fullerene C_60_ was isolated by precipitation in anhydrous diethyl ether (yield 84%). The light brown precipitate was separated by centrifugation and dried under a vacuum at 40 °C. Next, polycyclosulfated fullerene C_60_ was hydrolyzed for 12 h by adding bidistilled water to it. The resulting dark brown precipitate of polyhydroxylated fullerene was separated in a centrifuge and dried in vacuum at 40 °C (yield 93%). Next, fullerenol was mixed with oleum and stirred for 24 h under an inert N_2_ atmosphere. The resulting PHSF was isolated by precipitation in anhydrous diethyl ether. To prevent residual contamination with sulfuric acid, PHSF was additionally washed three times with anhydrous diethyl ether and twice with a 2:1 mixture of anhydrous diethyl ether/acetonitrile. The resulting precipitate was centrifuged and dried in a vacuum at 40 °C (yield 81%).

Fullerene functionalization was confirmed by FTIR-ATR, elemental analysis, and EDX. FTIR-ATR (ν, cm^−1^): 3413 (OH), 1376, 1218, (S=O), 1149, 1032, 959 (SO_3_^−^), 566 (C_60_) cm^−1^. Anal. (element, wt.%) Calcd for C_60_H_12_O_30_S_6_: C, 51.28; H, 0.85; O, 34.19; S 13.68. Found: C, 48.67; H, 2.42; S, 11.06 and EDX C, 51.44; O, 34.15; S, 14.41. The PHSF obtained was used to form fullerene-containing PEMs.

#### 2.2.4. Preparation of Composite Membranes

The composite membranes were obtained from the PVT and PHSF solutions using the solution casting method. The PVT solution was prepared by dissolving 0.3 g of polymer in 2.7 g of MP. PHSF was dissolved in MP for 2 h to obtain a brown dispersion. The sulfofullerene weight for different membranes was 0.5, 2, and 5 wt.% relative to the polymer weight. Next, PHSF was added to the PVT solution at a rate of 0.5 mL/min, and the mixture was stirred for 2 h on a magnetic stirrer. After this, polyvinyl alcohol (PVA) and oxalic acid (OA) were added to the reaction mixture. The initial PVA and OA samples were also dissolved in MP. The mass ratio of PVT-PHSF to PVA-OA in the reaction mixture was 1:0.5. The mixture was stirred for 2 h at a temperature of 70 °C and then applied in a thin layer by casting onto teflon substrates. Next, to obtain the membranes, they were dried for 12 h in air, then for 3 h in a deep vacuum at a temperature of 80 °C. The average thickness of the obtained composite membranes containing 0.5, 2, and 5 wt.% PHSF was 274, 287, and 361 μm.

## 3. Results and Discussion

### 3.1. Preparation of PVT-PHSF Composite Membranes

**Figure 2:**
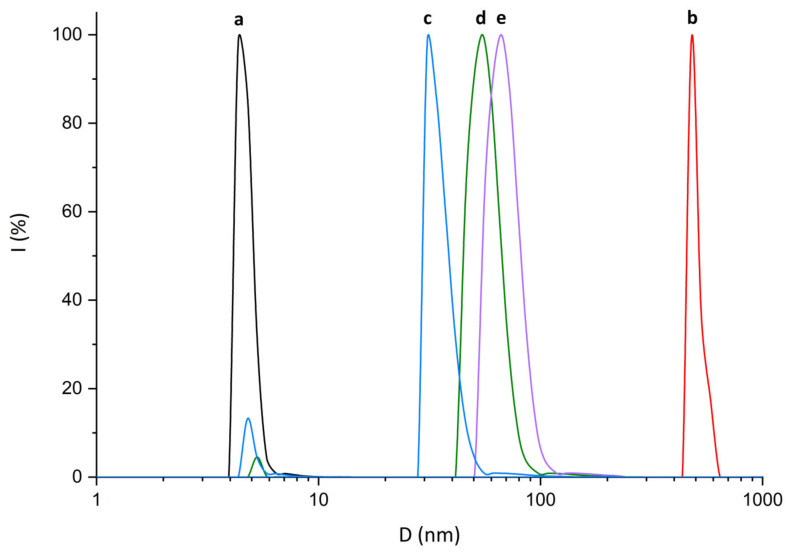
Histogram of the distribution of scattering particles over hydrodynamic diameters for solutions PVT (a), PHSF (b), reaction mixture PVT-PHSF 0.5 wt.% (c), 2 wt.% (d), and 5 wt.% (e).

To obtain fullerene-containing membranes, at the first stage, solutions of PVT and PHSF were prepared in MP. It is known that fullerenes and their derivatives tend to aggregate, and the size of the resulting nanoparticles significantly depends on the type of solvent and the method used to mix the solutions [58]. Therefore, the solutions were mixed by gradually adding PHSF to PVT at a constant rate of 0.5 mL/min. Measurement of hydrodynamic diameters by dynamic light scattering showed that the PVT in MP is in the form of macromolecular coils about 5 nm in size. The average size of the solvated PHSF particles in the same solvent was 477 nm. It is assumed that the self-aggregation of sulfofulerene molecules is caused by electrostatic interactions between functional groups [28,38,59]. Figure 2 shows that, with an increase in the mass fraction of PHSF to 0.5, 2, and 5 wt.% in the reaction mixtures, an increase in average hydrodynamic diameter of the particles to 30, 55, and 67 nm, respectively. In the reaction mixture containing 0.5 wt.% PHSF, a fast PVT mode is observed. This indicates the presence of a free polymer. The original polymer signal was no longer observed when the PHSF loading was increased to 5 wt.%, indicating intermolecular interactions between the entire PVT and PHSF. This results in the stabilization of the carbon nanoparticles by the PVT polymer matrix.

The high complexing ability of PVT is due to the presence of an unshared pair of electrons at the pyridine nitrogen atom in the triazole ring. Due to this, triazole fragments of PVT exhibit basic properties [63]. In PHSF, the covalent attachment of functional groups to the C_60_ carbon framework results in the transfer of electron density from sulfonic groups to the conjugated fullerene system. This increases the mobility of hydrogen atoms in sulfofullerene, which provides it with acidic properties comparable to benzenesulfonic acid [30,64].

When the solutions are mixed, a donor-acceptor interaction occurs between the “pyridine” nitrogen atom of the triazole ring in PVT and the sulfonic acid groups of PHSF. Interfacial interaction leads to the formation of acid–base complexes, which is accompanied by a decrease in the size of PHSF nanoparticles. An increase in the PHSF content leads to an increase in the diameter of macromolecular coils up to 30–70 nm, indicating intermolecular cross-linking of individual PVT macromolecules via carbon nanoparticles acting as a crosslinking agent. This indicates the effective interaction of sulfonic groups with triazole fragments and the stabilization of PHSF nanoparticles by a PVT polymer matrix.

In the second stage of composite PEM formation, PVA and oxalic acid were added to the resulting PVT-PHSF reaction mixture to form a cross-linked polymer matrix. An intermolecular dehydration reaction between the hydroxyl groups of PVA and the carboxyl groups of OA results in the formation of a three-dimensional polymer network of cross-linked PVA. As a result, linear PVT containing PHSF and cross-linked PVA form a single semi-interpenetrating polymer network structure.

**Figure 3:**
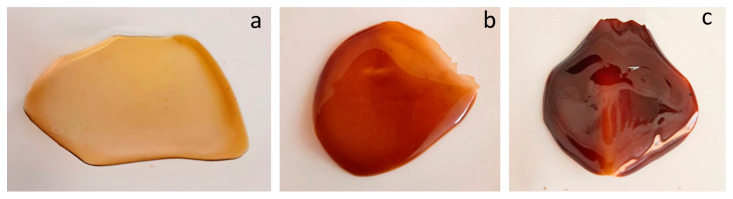
Characteristic appearance of composite PEM PVT–PHSF 0.5 wt.% (**a**), 2 wt.% (**b**), and 5 wt.% (**c**).

The resulting reaction mixture of PVT-PHSF:PVA:OA, with a ratio of 1:1:0.5, was thoroughly mixed and poured onto a substrate, then dried in a vacuum at 80 °C. In this way, the elastic nanocomposite PEM containing 0.5%, 2%, and 5 wt.% sulfofullerene was obtained (Figure 3).

### 3.2. FTIR Spectroscopy

**Figure 4:**
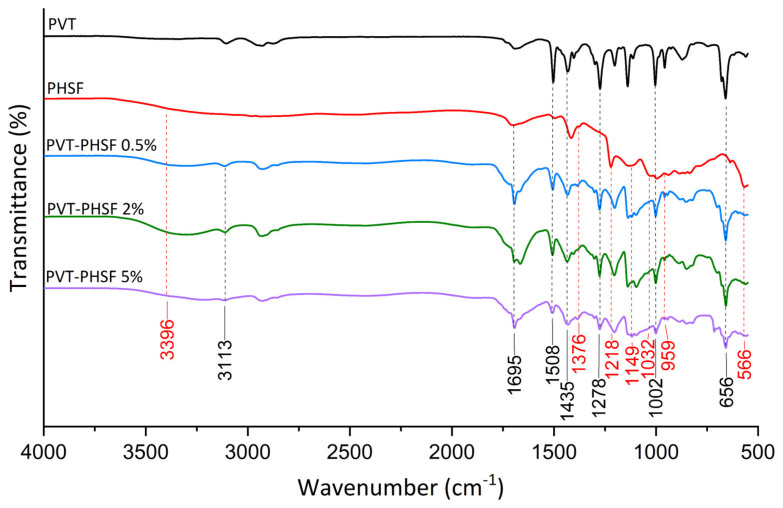
Comparative FTIR spectra of PVT, PHSF and composite PEM PVT-PHSF 0.5% wt.%, 2 wt.%, and 5 wt.%.

The FTIR-ATR spectra of nanocomposite PEMs with different sulfofullerene content are shown in Figure 4. The spectra show small shifts in the absorption bands at 1504–1508 (C=N), 1432–1435 (C–N), 1277–1278 (N–N), 1002 (C-H), and 658–660 (C–N) cm^−1^, which are characteristic of the stretching and deformation vibrations of the PVT ring. The absorption bands in the region of 1695 (C=O) cm^−1^ are attributed to the carbonyl group in cross-linked PVA. In the region of 3396–3413 (OH), 1376, 1218 (S=O), 1149, 1032 (SO_3_^−^), 566 (C_60_) cm^−1^, weak and broadened signals characteristic of the hydroxyl and sulfonic groups of PHSF are observed. The weak intensity of these signals is associated with a low sulfofullerene content, and correlates with changes in the composition of nanocomposite PEMs [65]. The broad bands detected in the region of 3113–3115 (N–H) cm^−1^ indicate the protonation of the triazole ring and the formation of an acid–base complex of PVT with PHSF, and are consistent with data from other studies [30,42,45,66]. The IR spectroscopy results of the chemical structure of composite membranes confirm the stabilization of sulfofullerenes in the PVT polymer matrix through an acid–base interaction.

### 3.3. Thermal Analysis

**Figure 5:**
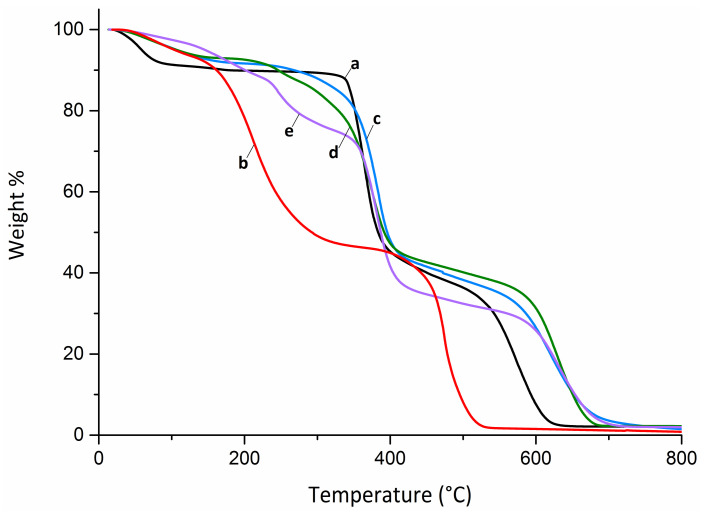
TGA curves for PVT (a), PHSF (b), composite PEM PVT-PHSF 0.5 wt.% (c), 2 wt.% (d), and 5 wt.% (e).

The thermal stability of proton-conducting membranes under fuel cell operating conditions at various temperatures is one of the key characteristics. The thermal-oxidative degradation of the synthesized PVT-PHSF nanocomposite membranes was studied using thermogravimetric analysis (TGA) under air atmosphere, as shown in Figure 5.

Thermogravimetric analysis results showed that PVT exhibits thermal stability up to 330 °C. PVT-PHSF nanocomposite membranes undergo a three-step degradation process. In the temperature range of 50–150 °C, a mass loss of 8% is observed, which is due to the higher content of bound water due to the acid–base interactions between the components. For nanocomposite membranes containing 0.5, 2, and 5 wt.% PHSF, the onset of thermal-oxidative degradation was observed at temperatures of 240, 245, and 303 °C, respectively. This indicates a decrease in the thermal stability of the composite membranes due to the low thermal stability of PHSF (180 °C) and the functional groups decomposition. Mass loss occurs above 401 °C to 595 °C due to the destruction of the main polymer chain and the sublimation of fullerene C_60_ [67]. The ash residue for all samples is 1–2.5%.

### 3.4. Microstructural Analysis

**Figure 6:**
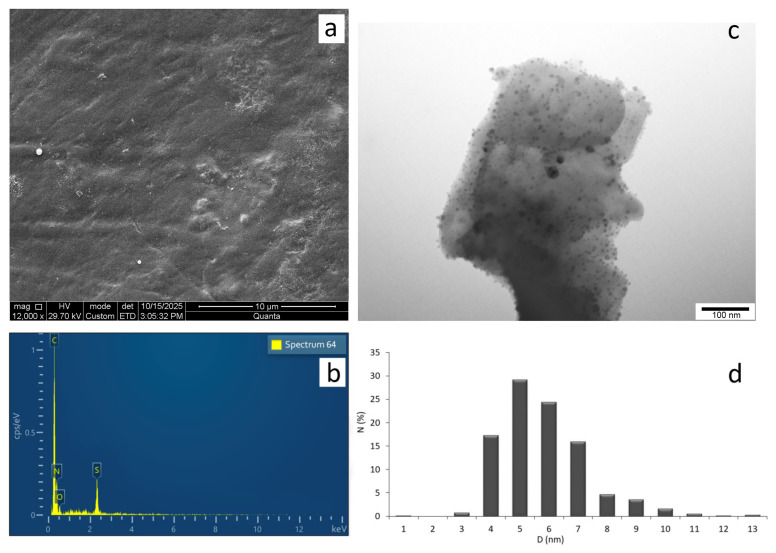
SEM (**a**) and TEM (**c**) micrographs, EDX results (**b**), and nanoparticle size distribution histogram (**d**) of the nanocomposite PEM PVT-PHSF 2%.

To determine the nature of the distribution of PHSF nanoparticles in the structure of the PVT polymer matrix, the fullerene-containing PEMs obtained were studied using electron microscopy methods (TEM, SEM). Figure 6 shows the micrographs of a PVT-PHSF nanocomposite membrane containing 2 wt.% sulfofullerene.

The SEM analysis results of the membranes show a uniform surface morphology for the composite PEM (Figure 6a). The EDX analysis of the membrane surface indicated the elemental composition characteristic of the PVT and PVA polymer matrices (Figure 6b). The presence of sulfur atoms indicates the significant presence of sulfonic acid groups on the PHSF membrane surface, which is a prerequisite for efficient proton transport through the PEM nanocomposite.

The shape, size, and distribution pattern of PHSF nanoparticles in the structure of semi-interpenetrating PEM composite networks was determined using transmission electron microscopy. The TEM micrograph (Figure 6c) clearly shows that isolated electron-contrast PHSF nanoparticles are uniformly distributed in the PVP polymer matrix and have predominantly a regular spherical shape. The sizes of carbon nanoparticles vary within a narrow range of 4 to 10 nm Figure 6d.

The presented electron microscopy results demonstrate a uniform structure and a smooth surface of the composite PEMs. Formation of membranes using MP as a solvent ensures uniform distribution of nanosized PHSF particles and reduces possible aggregation processes due to better chemical affinity between the components. These improvements may also be due to chemical interactions between the triazole fragments of PVT and the functional groups of the functionalized fullerene.

### 3.5. Oxidative Stability

An important characteristic of PEM is oxidative stability. When exposed to Fenton’s reagent for 3 h, composite membranes containing 0.5, 2, and 5 wt.% PHSF demonstrated weight losses of 16.3, 12.2, and 8.7%, respectively. Notably, the oxidative stability of the membranes increased significantly with increasing PHSF content. This is likely due to the scavenging of radicals by PHSF nanoparticles. As a result, the composite membrane’s structure is less susceptible to degradation by Fenton’s reagent. Thus, it can be concluded that the resulting composite membranes exhibited good oxidative stability.

### 3.6. Proton Conductivity

Proton conductivity is a key characteristic that determines the PEM efficiency. It is known that proton transfer in PVT-based systems is ensured through the formation of hydrogen bonds between adjacent triazole units [68]. In this case, proton transport is predominantly carried out by the Grotthuss mechanism, the speed of which is significantly higher in comparison with the vehicular mechanism [69,70]. The mechanisms of proton transport in composite systems are more complex than in conventional polymeric PEMs. This is due to the fact that nanoparticles intercalated into PEM affect both the structure of the polymer matrix and the formation of proton-conducting channels. In this case, the dispersion and nature of the particle distribution in the polymer matrix structure play a decisive role.

The results of impedance spectroscopy of the synthesized fullerene-containing PEMs showed an increase in proton conductivity of almost one order of magnitude in the temperature range of 25–80 °C at a relative humidity of 12%. A significant increase in the proton conductivity, from 0.95 to 1.67 mS/cm, was observed with increasing PHSF loading. The highest proton conductivity was demonstrated by a PEM composite containing 5 wt.% PHSF. While the proton conductivity of the commercial Nafion 117 membrane under these conditions is 1.2 mS/cm.

**Figure 7:**
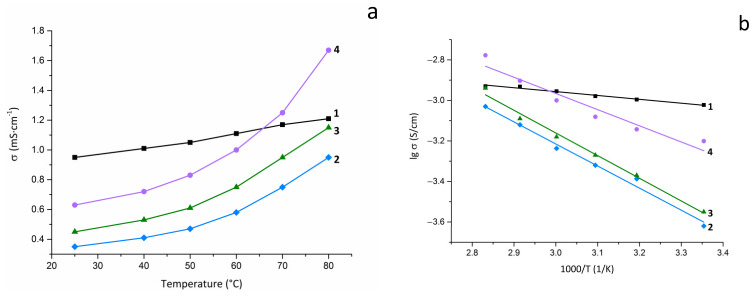
Temperature dependence of proton conductivity (**a**) and Arrhenius coordinates dependence (**b**) for Nafion 117 (1), composite PEM PVT-PHSF 0.5 wt.% (2), 2 wt.% (3), and 5 wt.% (4).

Based on the temperature dependence of the conductivity, the activation energy (Ea) values of proton transport were calculated for all studied membranes. It was found that, with increasing PHSF loading in composite membranes, the Ea decreases linearly from 0.46 to 0.32 eV (Figure 7). It is known that in PHSF nanoparticles, the functional groups are located close enough (about 2.5 Å) to allow proton jumping. It is likely that the acid–base interaction of PHSF and PVT reduces the distance between their functional groups, which facilitates the proton jump between them via the Grotthuss mechanism. Increasing sulfofullerene loading increases the contribution of this process to the overall proton conductivity, and Ea decreases. Thus, PHSFs participate in the proton transfer process, acting both as a proton-conducting phase and as a protons source [29].

As can be seen from the data in Table 1, the inclusion of PHSF in the polymer matrix leads to an increase in water uptake by 10–20%. Water absorption was accompanied by an increase in the swelling of the composite membranes proportionally to the increase in the PHSF content. Linear dimensions increased by 7–13%, and thickness by 11–32%. Acid leaching tests showed that the PHSF leaching rate from the synthesized composite membranes ranged from 1.3 to 5.8%, depending on the loading. This may be due to the dissolution of PHSF nanoparticles located on the membrane surface. However, the low acid leaching results suggest that PHSF internally leaks out of the membrane only slightly.

The high hydrophilicity of carbon nanoparticles and the polymer matrix help retain additional moisture in PEM. In this case, a sufficient number of hydrogen bonds are formed between water molecules and the functional groups of PHSF and PVT, which transfer protons via hopping and vehicle mechanisms. Due to this, fullerene-containing PEMs exhibit high proton conductivity at low humidity.

Considering that the acid–base interaction between PVT and PHSF is only possible at the phase boundary, we can assume that an increase in the dispersion of particles leads to an increase in the interphase interaction area. As previously shown, the nanosized PHSF particles (4–10 nm) are evenly distributed throughout the membrane structure. The high dispersion of PHSF nanoparticles ensures the most effective interphase interaction and the formation of additional proton-conducting channels in the nanocomposite PEM structure. Thus, the acid–base interaction between PHSF and PVT not only provides effective carbon nanoparticles stabilization but also leads to an increase in proton conductivity.

A comprehensive analysis of the obtained results suggests that proton transfer in synthesized fullerene-containing PEMs can occur through several pathways: (1) via protonated and unprotonated triazole rings, (2) via triazole rings and PHSF sulfo groups, and (3) via hydrogen bonding between water molecules and PHSF or PVT functional groups.

## 4. Conclusions

In this work, PVT-based nanocomposite PEMs containing 0.5, 2, and 5 wt.% of polyhydroxysulfonated fullerene were obtained for the first time. The introduction of functionalized fullerene PHSF into the PVT matrix increases water uptake and the number of proton-conducting channels in the membranes. The method used for forming membranes with polymer semi-interpenetrating networks allows obtaining nanocomposite PEMs with a uniform distribution of carbon nanoparticles up to 10 nm in size. The effective stabilization and high dispersion of PHSF nanoparticles lead to the formation of additional proton-conducting channels in the membrane structure. The impedance spectroscopy results demonstrate a significant increase in proton conductivity with increasing PHSF content, indicating the potential of the new nanocomposite PEMs. A discussion of possible proton transport pathways allows us to conclude that the introduction of functionalized fullerene PHSF affects both proton transport mechanisms (hopping and vehicle mechanisms) and the physical parameters of the PEM (water uptake). The synthesized fullerene-containing PEM PVT-PHSF exhibits high proton conductivity under low humidity conditions and is promising for use in fuel cells.

## Figures and Tables

**Table 1:** Proton conductivity, Ea, and water uptake of the PVT-PHSF nanocomposite membranes and Nafion 117.

Membranes	Proton Conductivity mS/cm (at 80 °C)	E_a_, eV	WU, %
Nafion 117	1.21	0.22	18
PVT-PHSF 0.5%	0.95	0.46	21
PVT-PHSF 2%	1.15	0.43	35
PVT-PHSF 5%	1.67	0.32	42

## Data Availability

The original contributions presented in this study are included in the article. Further inquiries can be directed to the corresponding author.

## Abbreviations

The following abbreviations are used in this manuscript:

PEM	Proton exchange membrane
FC	Fuel cell
PHSF	Polyhydroxysulfonated fullerene
IEC	Ion exchange capacity
VT	1-vinyl-1,2,4-triazole
PVT	Poly-1-vinyl-1,2,4-triazole
MP	1-methyl-2-pyrrolidone
FTIR-ATR	Fourier transform infrared with attenuated total reflectance
PVA	Polyvinyl alcohol
DLS	Dynamic light scattering
DMF	Dimethylformamide
TEM	Transmission electron microscopy
SEM	Scanning electron microscopy
